# Mediterranean Diet Patterns Are Positively Associated with Maximal Fat Oxidation and VO_2_max in Young Adults: The Mediating Role of Leptin

**DOI:** 10.3390/nu17111901

**Published:** 2025-05-31

**Authors:** Pablo Santiago-Arriaza, Juan Corral-Pérez, Daniel Velázquez-Díaz, Alejandro Pérez-Bey, María Rebollo-Ramos, Alberto Marín-Galindo, Adrián Montes-de-Oca-García, Miguel Ángel Rosety-Rodríguez, Cristina Casals, Jesús G. Ponce-González

**Affiliations:** 1 ExPhy Research Group, Department of Physical Education, Instituto de Investigación e Innovación Biomédica de Cádiz (INiBICA), Universidad de Cádiz, 11519 Cádiz, Spain; pablo.santiagoarriaza@mail.uca.es (P.S.-A.); daniel.velazquez@uca.es (D.V.-D.); alberto.marin@uca.es (A.M.-G.); adrian.montesdeoca@uca.es (A.M.-d.-O.-G.); cristina.casals@uca.es (C.C.); 2 School of Health Sciences, International University of La Rioja, 26001 Logroño, Spain; 3 GALENO Research Group, Department of Physical Education, Faculty of Education Sciences, University of Cádiz, Puerto Real, 11519 Cádiz, Spain; alejandro.perezperez@uca.es; 4 Move-It Research Group, Department of Physical Education, Instituto de Investigación e Innovación Biomédica de Cádiz (INiBICA), Universidad de Cádiz, 11519 Cádiz, Spain; maria.rebollo@uca.es (M.R.-R.); miguelangel.rosety@uca.es (M.Á.R.-R.)

**Keywords:** MFO, metabolic flexibility, nutrition, obesity, health

## Abstract

**Background:** Adherence to the Mediterranean diet (MedDiet) offers multiple metabolic benefits. However, its relationship with maximal fat oxidation (MFO) and cardiorespiratory fitness (VO_2_max), alongside the potential mediating role of leptin, remains underexplored in young adults. **Objective:** The objective was to investigate the associations between MedDiet adherence and the body mass index (BMI), MFO, and VO_2_max and to evaluate whether leptin mediates these relationships. **Methods:** Sixty-five young adults (*n* = 23 women), aged 18–38, were assessed for body composition, MedDiet adherence (14-Item Mediterranean Diet Adherence Screener), MFO, and VO_2_max through indirect calorimetry. Plasma leptin concentrations were measured in fasting conditions. Multiple linear regression models were performed, adjusting for sex, age, and both. Mediation analyses were conducted. **Results:** Higher MedDiet adherence was significantly associated with lower BMI (β = −0.339, *p* = 0.006) and leptin values (β = 0.284, *p* = 0.022) and higher absolute MFO (β = 0.338, *p* = 0.006) and VO_2_max values (β = 0.462, *p* < 0.001). These associations remained significant in all models except BMI and leptin when adjusted for sex and sex and age. Leptin was positively associated with the BMI (β = 0.550, *p* < 0.001) and inversely associated with absolute MFO (β = −0.650, *p* < 0.001) in all models. There was a trend in the association between leptin and VO_2_max (β = −0.233, *p* = 0.061) only in the unadjusted model. Mediation analysis revealed that the leptin levels significantly mediated the associations between MedDiet adherence and BMI (β = −0.358, 95% CI [−0.677, −0.077]) and VO_2_max (β = 1.043, 95% CI [0.280, 1.833]). **Conclusions:** MedDiet adherence is associated with a lower BMI and higher MFO and VO_2_max in young adults. Our findings further suggest that leptin plays a mediating role in how MedDiet adherence influences the BMI and VO_2_max.

## 1. Introduction

Metabolic health is closely linked to the body’s capacity to regulate substrate utilization in response to varying energy demands. A reduced capacity for maximal fat oxidation (MFO) [1,2] and lower maximal oxygen consumption (VO_2_max) [3] have been identified as indicators of metabolic inefficiency. These parameters are often used to characterize metabolic inflexibility, a condition defined by a limited ability to switch between fuel sources such as fats and carbohydrates [1]. MFO in particular has been proposed as a functional marker of metabolic flexibility [4,5,6], and lower fat oxidation rates during physical activity have been associated with greater cardiometabolic risk, including insulin resistance and excessive weight gain [4,7]. Investigating lifestyle-related factors associated with MFO and VO_2_max may contribute to a better understanding of physiological profiles linked to metabolic function.

Dietary patterns have been increasingly studied in relation to metabolic flexibility. Among them, the Mediterranean diet (MedDiet), characterized by high consumption of fruits, vegetables, whole grains, nuts, and olive oil, along with moderate intake of fish and wine, is widely recognized for its association with favorable health indicators [8]. This dietary model has been linked to lower levels of inflammation, showing that a higher adeherence to the MedDiet reduces letpin [9], leads to healthier cardiovascular profiles by reducing the risk of coronary heart disease, stroke, myocardial infarction, and overall cardiovascular disease [10], and better glycemic regulation, leading to greater reductions in HbA1c, fasting glucose, and fasting insulin [10,11]. However, despite the broad range of reported associations, the relationship between MedDiet adherence and MFO remains insufficiently studied, representing a relevant gap in the literature.

Moreover, emerging evidence suggests a potential relationship between the MedDiet and VO_2_max. While a systematic review and meta-analysis indicates a general association between greater MedDiet adherence and better physical fitness across various adult populations [12], research specifically for young adults remains inconsistent. Although research specifically examining this association is still limited, one cross-sectional study in young adults reported no significant correlation between overall MedDiet adherence and VO_2_max. However, nut consumption, a key component of the MedDiet, was positively associated with higher VO_2_max levels in the same study [12]. Conversely, studies in other populations, such as obese patients with hypertrophic cardiomyopathy, have shown significant VO_2_max improvements when the Mediterranean diet is combined with exercise [13], and similar benefits have been noted in athletes [14]. This variability highlights the need for further investigation into the specific conditions and populations where MedDiet adherence most strongly influences cardiorespiratory fitness.

Leptin may contribute to the relationship between MedDiet adherence and physiological parameters such as MFO and VO_2_max. This adipocyte-derived hormone plays a key role in energy homeostasis and fat metabolism. Experimental studies suggest that leptin could influence fat oxidation through mechanisms involving mitochondrial function and substrate preference, although the exact pathways remain under investigation [15]. However, evidence in humans remains limited and inconsistent, particularly regarding its relationship with aerobic capacity [16]. Moreover, leptin levels are influenced by dietary patterns and adiposity, making it a plausible mediator of dietary effects on metabolic outcomes [17]. Based on this rationale, the present study explores whether leptin mediates the relationship between MedDiet adherence and key indicators such as the body mass index (BMI), MFO, and VO_2_max in young adults.

To address this knowledge gap, the present study examines the associations between adherence to the MedDiet pattern and the BMI, absolute and relative MFO capacity, VO_2_max, and leptin levels. Moreover, we aim to determine whether leptin mediates these associations, offering novel insights into the mechanisms linking adherence to the MedDiet pattern to improvements in BMI, metabolic flexibility, and cardiorespiratory fitness.

## 2. Materials and Methods

### 2.1. Study Design

The present investigation represents a subsample derived from the cross-sectional study of the NutAF Project (Nutritional Habits and Physical Activity Levels in Adults) [18,19,20,21,22] within a cohort of adults in the province of Cádiz (Spain). Data collection occurred between January 2016 and June 2017. The study received ethical approval from the Research Ethics Committee of Cádiz (Hospital Puerta del Mar), according to the Declaration of Helsinki. All testing was conducted in the Laboratory of Physical Activity and Exercise at the University of Cádiz (Puerto Real, Cádiz, Spain). Written informed consent was obtained from all participants following a thorough explanation of the study’s objectives and methodologies and any potential risks inherent in the measurement procedures.

### 2.2. Participants

From the total sample of the NutAF study (*n* = 79), a cohort of 65 participants (*n* = 23 women; mean age = 22.35 ± 4.30 years) who had complete data for all variables analyzed in the present article were selected for analysis. All participants met the following inclusion and exclusion criteria: (1) age between 18 and 45 years; (2) BMI between 18.5 and 40 kg·m⁻^2^; (3) stable body weight (±2 kg) over the previous six months; (4) non-smoker; (5) no current or previous diagnosis of hypertension or cardiovascular disease; (6) no recent injuries or medical conditions interfering with physical testing; and (7) no adherence to specific or restrictive diets, including weight loss regimens, during the past six months. These inclusion and exclusion criteria were selected because it has been observed in the scientific literature that changes in body mass induced by exercise and diet can modify the fat oxidation capacity in young adults [23].

### 2.3. Procedure

All measurements were performed on a single day under standardized conditions in the morning (from 8:00 a.m. until 11:30 a.m.). Participants arrived at the laboratory in a fasted state (≥8 h) and were instructed to maintain their usual dietary and hydration habits while avoiding alcohol, caffeine, and intense physical activity in the 24 h prior to testing. Upon arrival, participants first completed the validated 14-item Mediterranean diet adherence questionnaire. Then, venous blood samples were collected to determine the plasma leptin concentrations. Subsequently, anthropometric and body composition measurements were performed using bioelectrical impedance analysis. Participants then completed the validated 14-item Mediterranean diet adherence questionnaire. Finally, an incremental exercise protocol on a cycle ergometer was used to determine maximal fat oxidation (MFO) by indirect calorimetry, followed by a previously validated VO_2_max test [24]. All procedures were conducted in a controlled environment (21 ± 1 °C and 50 ± 2% relative humidity), following standardized protocols previously applied in the NUTAF project.

#### 2.3.1. Evaluation of Adherence to the Mediterranean Diet (MedDiet)

Adherence to the MedDiet was assessed using a validated 14-item Mediterranean diet adherence questionnaire [25] specifically devised for assessing MedDiet adherence in the PREDIMED study and previously validated in the Spanish population [26,27]. Each item was assigned a score of 0 or 1, with a maximum score of 14 points reflecting optimal adherence. The resulting score was classified into two categories: low adherence to the MedDiet (range: 0–7) and high adherence to the MedDiet (range: 8–14) [28]. The questionnaire was administered by trained research staff through face-to-face interviews to ensure consistency and accuracy in responses.

#### 2.3.2. Biochemical Analysis

Fasting blood samples were collected via venipuncture and stored in Vacutainer tubes, with some containing EDTA anticoagulant. These samples were then subjected to centrifugation (2500 rpm, 15 min, 4 °C) to separate the plasma and stored at −80 °C until further analysis. Plasma leptin concentrations were determined using a MILLIPLEX^®^ MAP Human Metabolic Hormone Magnetic Panel (HMHEMAG-34K, Millipore Sigma, Burlington, MA, USA) and the Luminex^®^ 200™ system (Luminex Corp., Austin, TX, USA), in accordance with the manufacturer’s instructions. The intra-assay coefficients of variation were <15%.

#### 2.3.3. Body Composition and Anthropometry

Body composition was assessed using bioelectrical impedance analysis, measuring body mass and total muscle mass with a Tanita MC-780MA multifrequency 8-electrode model (Tanita Corp, Tokyo, Japan). Standardized protocols were followed [5], including urination prior to the assessment and refraining from wearing metal objects. Anthropometric measurements were taken with the participants being barefoot and wearing light clothing. Height was measured in a standing position using a stadiometer (SECA 225; range: 60–200 cm; precision: 1 mm). Finally, the BMI was calculated by dividing the body mass (kg) by the height squared (m^2^). For the purposes of this study, the participants were divided into two groups based on their BMIs: normal weight (BMI: 18.5–24.9 kg/m^2^) and overweight or obese (BMI ≥ 25.0 kg/m^2^), following World Health Organization (WHO) classification [29].

#### 2.3.4. Basal Metabolism

The study of the resting metabolic rate focused on oxygen consumption (VO_2_), carbon dioxide production (VCO_2_), and the respiratory exchange ratio (RER). Participants were instructed to remain in a supine position for 30 min, adhering to specific protocols for measuring basal metabolic rate in healthy adults [30]. Measurements were conducted via indirect calorimetry using a gas analyzer (Jaeger MasterScreen CPX^®^ CareFusion, San Diego, CA, USA), which underwent daily calibration. During the assessment, gas analyzer readings were captured on a breath-by-breath basis and averaged every 20 s. The initial 5 min of data were excluded, with analysis performed on the subsequent 5 min, ensuring a coefficient of variation for VO_2_ and VCO_2_ not exceeding 15%. The average values of VO_2_ and VCO_2_ for the selected interval were then employed to estimate resting fat oxidation through an indirect equation [31]. This value was used as the first point on the fat oxidation curve.

#### 2.3.5. Maximal Fat Oxidation (MFO) and Maximal Oxygen Consumption Test (VO_2_max)

In order to assess MFO, VO_2,_ and VCO_2_, values were recorded during an incremental test performed on a cycle ergometer by utilizing a Jaeger MasterScreen CPX^®^ gas analyzer. This procedure was based on an adapted version of a previously validated protocol [32]. The test started with a load of 30 W, which was progressively increased every 3 min until the respiratory exchange ratio (RER) reached 1.0. After 5 min for recovery, the load was further increased by 30 W per minute until exhaustion, beginning in the last load of the MFO test, which allowed for measurement of VO_2_max. For the obese participants, the initial load and load increments were set at 15 W. A constant cadence of 60–80 revolutions per minute was maintained, with continuous heart rate monitoring.

Fat oxidation for each stage was calculated by averaging the VO_2_ and VCO_2_ values from the last minute of each stage using Frayn’s equation [31]. The average VO_2_ was also used to calculate the percentage of maximal VO_2_ achieved. A polynomial curve was then fitted to the data for each participant, enabling comparisons of absolute MFO and MFO relativized by body mass (MFO-BM) and total muscle mass.

### 2.4. Statistical Analysis

Statistical analyses were conducted using SPSS version 29 (SPSS Inc., IBM, Armonk, NY, USA). A significance level of 95% (*p* ≤ 0.05) was established for all analyses. The normality of the distributions was assessed using the Kolmogorov–Smirnov test. Variables that deviated from a normal distribution were subjected to natural logarithmic transformation prior to further analyses. To compare the general characteristics of the sample based on BMI category (normal weight vs. overweight or obese) and adherence to the Mediterranean Diet (low vs. high), independent samples *t*-tests were conducted. Effect sizes were calculated using Cohen’s d and interpreted according to conventional thresholds (small: ≥0.2; medium: ≥0.5; large: ≥0.8).

Linear regression models were employed to explore the associations between adherence to the MedDiet as the independent variable and the BMI, absolute MFO, MFO per body mass (MFO-BM), MFO per total muscle mass (MFO-MM), VO_2_max, and leptin concentration as dependent variables. Four distinct linear regression models were evaluated: Model 0 (unadjusted), Model 1 (adjusted for sex), Model 2 (adjusted for age), and Model 3 (adjusted for sex and age). Moreover, linear regression models were employed to explore the associations between leptin as the independent variable and the BMI, absolute MFO, MFO-BM, MFO-MM, and VO_2_max as dependent variables with the same models.

Additionally, to ascertain the potential influence of leptin concentrations on adherence to the MedDiet and their impact on the absolute MFO, MFO-BM, MFO-MM, and VO_2_max, mediation analyses were conducted with leptin as the mediating variable. The mediating variable was similarly adjusted in alignment with the dependent variable. Linear regression models were adjusted using bootstrapping methods and implemented through Hayes’ PROCESS macro for SPSS (Armonk, NY, USA), employing a resampling procedure of 10,000 bootstrap samples [33,34]. This methodology facilitates the computation of confidence intervals for mediator effects without requiring assumptions regarding data distribution. Thus, mediation analyses were performed to assess the role of leptin concentrations as a mediator between adherence to the MedDiet and several outcomes: BMI, absolute MFO, MFO-BM, and VO_2_max. The analysis evaluated the total effect (Equation (2)) and the direct effect (Equations (1), (3), and (3’)), based on unstandardized regression coefficients (B) and their corresponding significance levels. Specifically, Equation (1) regressed the independent variable (MedDiet adherence) on the mediator (log-transformed leptin concentrations). Equation (2) regressed the independent variable (MedDiet adherence) on each dependent variable (BMI, absolute MFO, MFO-BM, and VO_2_max). Equation (3) regressed both the mediator (leptin concentrations) and the independent variable (MedDiet adherence) on each dependent variable. Acknowledging that optimal mediation analysis typically benefits from larger samples (e.g., >200 participants), we employed 10,000 sample bootstrapping to strengthen the statistical inference despite our cohort of 65, as previously performed by other authors [18]. Subsequently, indirect effects were calculated, and their 95% confidence intervals (CIs) were estimated using bootstrapping procedures (10,000 resamples) [34,35], a non-parametric method that does not assume normal distribution of the indirect effects. Mediation was considered present when the bootstrap confidence interval for the indirect effect did not include zero.

## 3. Results

Table 1 presents the general characteristics of the study participants and their differences according to BMI category (normal weight vs. overweight or obese) and adherence to the MedDiet (low vs. high). Participants who were overweight or obese showed higher BMI and leptin levels and lower absolute and relative MFO and VO_2_max levels compared with the normal-weight individuals, with large effect sizes (Cohen’s d > 0.8). Similarly, those with low adherence to the MedDiet exhibited higher leptin, lower VO_2_max, and lower MFO levels (particularly per body weight MFO-BM), with moderate-to-large effects.

### 3.1. Associations of Mediterranean Diet Adherence with Body Mass Index, Maximal Fat Oxidation, VO_2_max, and Leptin Concentration

Table 2 presents the associations between adherence to the MedDiet and several metabolic indicators. Higher adherence to the MedDiet was significantly associated with lower BMI, higher MFO (absolute and relative), greater VO_2_max, and lower leptin levels in the unadjusted model. These associations remained significant after adjusting for sex. After adjusting for age or both sex and age, associations with MFO and VO_2_max persisted, while those with the BMI and leptin were attenuated and showed a trend (*p* < 0.1). Among the studied outcomes, VO_2_max and MFO-BM exhibited the strongest associations with MedDiet adherence, indicated by their higher standardized coefficients (β) and explained variance (R^2^) across models.

### 3.2. Associations of Leptin Levels with Body Mass Index, Maximal Fat Oxidation, and VO_2_max

Table 3 summarizes the associations between log-transformed leptin levels and key metabolic outcomes. Higher leptin concentrations were consistently associated with higher BMI and lower absolute MFO levels across all models, even after adjustments for sex and age. Associations with MFO-BM were significant in the unadjusted and sex-adjusted models and remained as a trend after adjusting for age and for both sex and age (*p* < 0.1). A similar trend was observed for VO_2_max in the unadjusted model, although this association did not reach statistical significance after adjustment.

### 3.3. Mediation Analyses: Leptin Concentration and Mediterranean Dietary Pattern with Absolute MFO, MFO Body Mass, and VO_2_max

**Figure 1:**
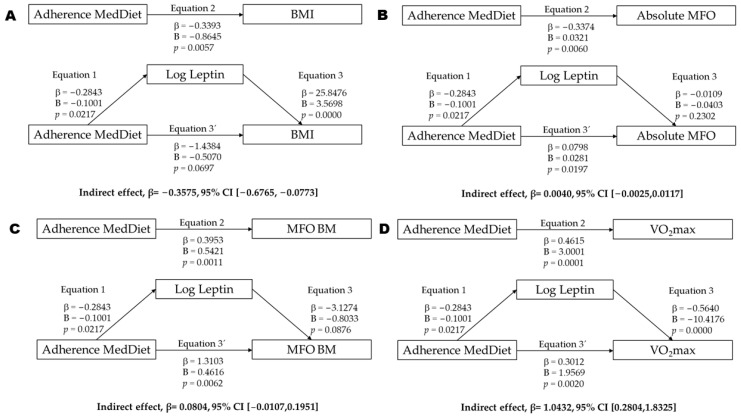
Mediation analysis of leptin concentration between adherence Mediterranean diet (MedDiet) and BMI (**A**), absolute MFO (**B**), MFO relativized by body mass (**C**), and VO_2_max (**D**). Abbreviations: *p* = *p* value; β = standardized regression coefficient; B = unstandardized regression coefficient; BMI = body mass index (kg/m^2^); MFO = maximal fat oxidation; Leptin = log-transformed leptin concentrations; VO_2_max = maximal oxygen uptake.

Mediation models were conducted to explore whether leptin mediates the association between MedDiet adherence and specific health-related outcomes (BMI, MFO, and VO_2_max; Figure 1). Leptin significantly mediated the relationship between adherence to the MedDiet and the BMI, with a significant indirect effect (β = −0.358, 95% CI [−0.677, −0.077]; Figure 1A) supported by significant paths from the MedDiet to leptin and from leptin to the BMI. In contrast, no mediation effect was found for absolute MFO (β = 0.004, 95% CI [−0.003, 0.012]; Figure 1B), as leptin was not significantly associated with this outcome. For MFO relative to body mass, the indirect effect approached significance (β = 0.080, 95% CI [−0.011, 0.195]; Figure 1C), with a significant direct association and a trend observed in the leptin-to-MFO path (*p* = 0.0876), suggesting possible partial mediation. Finally, a significant mediation effect was found for VO_2_max (β = 1.043, 95% CI [0.280, 1.833]; Figure 1D), with both the MedDiet-to-leptin and leptin-to-VO_2_max paths reaching statistical significance.

## 4. Discussion

### 4.1. Main Research Findings

The present study demonstrates that higher adherence to the MedDiet is associated with improved metabolic health in young adults. Our findings revealed significant associations between greater MedDiet adherence and lower BMI, higher MFO, greater VO_2_max, and lower leptin levels. Furthermore, our mediation analyses suggest that leptin may partially explain the observed associations between MedDiet adherence, body composition, and aerobic capacity. These findings collectively highlight the multifaceted benefits of the MedDiet and the potential role of leptin as a mediating factor.

### 4.2. Associations Between Adherence to the Mediterranean Diet and the Study Variables

In agreement with our results, there is previous evidence linking the MedDiet with reduced risk of obesity, as well as improved body composition and metabolic health [36,37,38,39]. This is likely attributable to the elevated intake of fiber-rich foods, healthy fats, and antioxidants inherent in the MedDiet, which foster satiety, mitigate systemic inflammation, and enhance insulin sensitivity while also being linked to higher levels of MFO, as evidenced in prior research [40]. Our results showed a consistent positive association between MFO and the MedDiet across all models, even after adjustments for sex and age. This finding suggests that individuals with greater adherence to this dietary pattern may exhibit an enhanced capacity to utilize fat as an energy substrate during submaximal exercise, suggesting improved metabolic flexibility.

Furthermore, this enhanced metabolic flexibility could be attributed to the improvements in metabolic efficiency resulting from the consumption of healthy fats (monounsaturated and polyunsaturated), which favor the utilization of fatty acids as an energy substrate [41]. The MedDiet is rich in monounsaturated fatty acids (MUFAs), primarily from olive oil, and polyunsaturated fatty acids (PUFAs), particularly omega-3s from fish and nuts. These healthy fats are known to enhance mitochondrial biogenesis and function, thereby improving the capacity for fat oxidation within cells [42]. For instance, MUFAs have been shown to upregulate genes involved in fatty acid transport and beta-oxidation [43]. Similarly, omega-3 fatty acids can influence peroxisome proliferator-activated receptors (PPARs), which are key regulators of lipid metabolism and mitochondrial function [44]. Additionally, it has also been reported that the anti-inflammatory and antioxidant effects of the MedDiet could enhance the fat oxidation capacity and mitochondrial function [45]. The abundance of antioxidants, such as polyphenols from fruits, vegetables, and olive oil, helps to reduce oxidative stress, which can otherwise impair mitochondrial integrity and reduce metabolic efficiency [46]. Chronic low-grade inflammation, often associated with Western diets, can also negatively impact insulin sensitivity and substrate utilization. Thus, the anti-inflammatory compounds in the MedDiet may directly foster a more efficient fat-burning state [46]. Hence, the implementation of this diet could be strategically advantageous if tailored to balance healthy fats with moderate carbohydrate consumption.

Moreover, these fiber-rich foods may modulate the composition and metabolism of the gut microbiota, thereby enhancing lipid metabolism [47] and stimulating the secretion of glucagon-like peptide-1 (GLP-1), which has been associated with increased fat oxidation [48].

Initially, adherence to the MedDiet was inversely associated with the leptin concentrations. While this may seem paradoxical, given that leptin is a hormone involved in appetite suppression and fat oxidation, it is important to note that leptin is primarily secreted by adipocytes, and its circulating levels are typically elevated in individuals with obesity due to leptin resistance. In this context, higher MedDiet adherence may reflect better metabolic health and lower adiposity, leading to reduced leptin levels. Moreover, adherence to the MedDiet may enhance leptin sensitivity, promoting more efficient regulation of energy homeostasis and fat metabolism during physical activity [49].

Therefore, a dietary pattern such as the MedDiet may be associated not only with lower leptin levels but also a more favorable hormonal profile potentially linked to leptin sensitivity. Higher levels of circulating leptin, particularly in the context of obesity, are often indicative of leptin resistance, where the brain and other tissues become less responsive to its signaling, perpetuating an energy imbalance [50]. The MedDiet’s emphasis on whole, unprocessed foods, a high fiber content, healthy fats, and antioxidants could contribute to a reduction in systemic inflammation and improved insulin sensitivity, both of which are strongly linked to enhanced leptin sensitivity [51]. Specifically, reduced adiposity resulting from MedDiet adherence would directly lead to lower leptin production, while improved inflammatory markers might enhance receptor function and leptin signaling pathways [51]. This possible hormonal pathway could help contextualize the observed associations between dietary habits, energy regulation, and indicators of metabolic and cardiovascular function.

Furthermore, dietary components characteristic of the Mediterranean pattern, such as monounsaturated fats from olive oil, polyunsaturated fats from nuts and fish, and bioactive compounds like polyphenols, may improve leptin signaling and reduce leptin resistance [52,53,54].

It is also notable that in this sample, leptin levels were negatively associated with MFO, particularly absolute MFO, across all models. This suggests that higher leptin concentrations, typically linked to higher fat mass, may reflect diminished fat oxidation capacity in less metabolically flexible individuals. This finding reinforces the role of the MedDiet in supporting a favorable hormonal and metabolic profile conducive to efficient fat metabolism.

### 4.3. Mediation Analysis: The Role of Leptin

Mediation analyses revealed that leptin significantly mediated the associations between MedDiet adherence and both the BMI and VO_2_max. These findings suggest that leptin may act as a key hormonal link between dietary patterns and metabolic outcomes, particularly in body composition and aerobic capacity. The inverse association between MedDiet adherence and leptin, combined with leptin’s positive relationship with the BMI and its trend with VO_2_max, supports a potential pathway in which healthier dietary habits are linked to lower leptin concentrations, which may be related to more favorable cardiorespiratory and adiposity profiles. Although no significant mediation effect was observed for MFO, a trend was noted for MFO relative to body mass, suggesting a possible partial mediating role of leptin in fat oxidation capacity. These results are in line with previous studies indicating that leptin resistance, often present in individuals with elevated leptin levels, impairs metabolic regulation and exercise capacity [55,56].

A recent study revealed that women with obesity and low fitness levels, irrespective of age, exhibit a higher MFO rate compared with their normal-weight counterparts with similar fitness levels [57]. This finding underscores the importance of adiposity as a determining factor in lipid oxidation processes. Moreover, it has been shown that individuals with elevated leptin levels have improved mitochondrial function and higher MFO compared with non-obese individuals [27].

In contrast, in this study, higher leptin levels were associated with lower absolute MFO across all models, suggesting that increased leptin may reflect greater adiposity or leptin resistance, rather than enhanced oxidative metabolism. Several studies suggest that leptin, through its role in regulating energy balance and fat metabolism, could influence fat oxidation rates [58]. Nevertheless, the trend to mediation observed for MFO relativized for muscle mass indicates that leptin may still play a compensatory role in modulating fat oxidation capacity when adjusted for the total muscle, potentially by influencing substrate utilization pathways at the muscular level. The possible mediating role of leptin in this context aligns with its established role in promoting oxidative capacity and energy expenditure.

Several studies have described an inverse association between leptin levels and cardiorespiratory fitness. In particular, Miller et al. [59] observed that plasma leptin concentrations were negatively correlated with VO_2_max in young adult men and women, independent of sex and body composition. This finding supports the hypothesis that higher leptin levels, often reflecting excess adiposity and potential leptin resistance, may impair aerobic performance. In line with this evidence, our study showed a negative trend in the association between leptin levels and VO_2_max and also found that leptin significantly mediated the relationship between MedDiet adherence and VO_2_max.

However, these findings should be interpreted with caution, as they reflect only a trend observed in the data and are based on a cross-sectional design, which precludes any inference of causality. Even so, our results point to a potential pathway through which adherence to the MedDiet could be associated with physiological fitness beyond traditional markers of adiposity, possibly involving leptin concentrations and sensitivity. While preliminary, these observations highlight the relevance of exploring hormonal factors that may contribute to the associations between dietary patterns and physical performance.

In addition, leptin was inversely associated with all MFO outcomes, which may reflect impaired metabolic flexibility due to leptin resistance. Therefore, higher leptin levels could promote an earlier reliance on glucose during physical effort, potentially limiting fat oxidation and contributing to a lower VO_2_max level. Conversely, MedDiet adherence was positively associated with MFO, suggesting that an improved fat oxidation capacity may represent an additional pathway through which dietary quality enhances aerobic performance, independent of changes in body composition.

Overall, these findings suggest that leptin may be involved in the association between MedDiet adherence, BMI, and VO_2_max, whereas its potential mediating role in fat oxidation appears to be limited.

### 4.4. Physiological and Practical Implications

The findings from the present study involve several important implications. Adherence to the MedDiet can be regarded as an effective strategy not only for improving body composition (reduction in BMI) but also for optimizing fat oxidation and cardiorespiratory performance, particularly in active populations or those at metabolic risk.

The findings underscore leptin’s pivotal role as a metabolic mediator. Enhancing leptin sensitivity through dietary interventions such as the MedDiet could serve as a key strategy for boosting energy efficiency. The mediation of VO_2_max by leptin suggests that the physiological benefits of the Mediterranean dietary pattern may be partially driven by endocrine modulation, particularly through improved leptin signaling involved in substrate utilization and aerobic performance. Although no significant mediation was observed for MFO outcomes, a tendency was noted for MFO relative to body mass, indicating a potential partial role of leptin in enhancing fat oxidation efficiency. These findings reinforce the idea that improvements in aerobic capacity associated with MedDiet adherence may not solely depend on changes in body composition but also underlying hormonal adaptations that influence energy metabolism during exercise.

Improvements in VO_2_max and MFO via leptin mediation suggest that the MedDiet could be particularly beneficial in training programs aimed at enhancing aerobic performance and utilizing fat as an energy substrate, with applications in both endurance sports and the prevention of metabolic disorders.

These findings could be of relevance to populations adhering to diets and patterns similar to the MedDiet, such as the Nordic diet [60], Dietary Approaches to Stop Hypertension (DASH) [61], and the Okinawa diet [62], by comparing their characteristics, cardiovascular health benefits, and relationships with longevity. It is notable how, despite cultural and geographical differences, all these diets share common healthy principles, such as high intake of fresh foods, low consumption of red meats, and a focus on healthy fats [63,64,65,66]. Therefore, examining their effects on maximal fat oxidation (MFO) may offer valuable insights into shared mechanisms promoting metabolic health. Future studies should investigate whether these alternative dietary patterns yield similar outcomes regarding leptin concentrations, cardiorespiratory fitness, and fat oxidation efficiency.

### 4.5. Study Limitations and Future Directions

Despite the significant findings, this study presents some limitations. Its cross-sectional design limits the ability to establish causal relationships. Although the sample size was sufficient to detect significant associations, it may have reduced sensitivity to identifying weaker effects in some outcomes. However, the use of a well-validated MedDiet adherence questionnaire enhances the reliability of dietary assessment, strengthening the interpretation of the associations observed. Additionally, the application of bootstrapped mediation analyses with 10,000 samples improves the robustness of the statistical inference.

Further studies are needed to determine whether these results apply to younger or older populations, individuals with diseases, or those who engage in regular physical activity.

A notable limitation of the present study is the unequal sex distribution within our cohort, with a lower proportion of women (*n* = 23) compared with men (*n* = 42). While we accounted for sex as a covariate in our statistical models to mitigate its potential influence on the associations, this imbalance might still affect the generalizability of our findings.

Moreover, body composition was measured using bioimpedance rather than more precise methods, such as dual-energy X-ray absorptiometry (DXA), which represents another limitation.

In turn, other potential mediating variables, such as physical activity, specific macronutrient composition, or inflammatory biomarkers, were not assessed, which could provide a more comprehensive understanding of underlying mechanisms. It is also worth noting that menstrual cycle phases and estrogen levels in women were not considered, despite their substantial influence on fat oxidation and insulin sensitivity [67].

However, the assessment of fat oxidation and cardiorespiratory capacity in a controlled environment, using laboratory tests and a gas analyzer, as well as the incorporation of biochemical parameters, would enhance the study design. Finally, future research should include a larger sample size, evaluation of other metabolic hormones, and exploration of longitudinal interventions.

## 5. Conclusions

In conclusion, MedDiet adherence is positively associated with MFO and VO_2_max, highlighting a novel link between dietary quality and substrate utilization in young adults. Furthermore, leptin emerges as a key hormonal mediator in the relationships between the MedDiet and both the BMI and VO_2_max, suggesting that the benefits of this dietary pattern may partially operate through endocrine pathways related to energy homeostasis. These findings reinforce the MedDiet as a valuable nutritional strategy not only for metabolic health promotion and prevention of adiposity-related conditions but also for optimizing fat metabolism and cardiorespiratory fitness in physically active populations.

Building on these insights, future research should aim to investigate the precise molecular mechanisms by which MedDiet components influence leptin secretion and sensitivity. Longitudinal intervention studies are also warranted to establish causality, exploring how sustained MedDiet adherence impacts leptin levels and subsequently affects changes in body composition and cardiorespiratory fitness over time. Additionally, examining these relationships in diverse populations, including those with pre-existing metabolic conditions or across a wider age range, would strengthen the generalizability and clinical applicability of our findings. 

## Figures and Tables

**Table 1:** General characteristics of the sample and differences by BMI category (normal weight vs. overweight or obese) and adherence to the Mediterranean Diet (MedDiet) category (low and high).

	All	NW	OW/OB	d BMI	*p* BMI	Low MD	High MD	d MD	*p* MD
Sex (Men/Women)	42/23	30/16	12/7			22/15	20/8		
Age (years)	22.35 ± 4.30	20.70 ± 2.15	26.37 ± 5.49	−1.644	**<0.001**	22.95 ± 5.16	21.57 ± 2.69	0.321	0.204
BMI (kg/m^2^)	24.78 ± 4.29	22.39 ± 1.54	30.54 ± 3.13	−3.843	**<0.001**	25.68 ± 5.15	23.59 ± 2.38	0.499	**0.049**
Absolute MFO (mg/min)	385.6 ± 159.9	411.9 ± 155.4	322.0 ± 156.2	0.578	**0.038**	354.0 ± 168.2	427.4 ± 140.3	−0.468	0.066
MFO body mass (mg/kg/min)	5.37 ± 2.31	6.11 ± 2.09	3.58 ± 1.80	1.262	**<0.001**	4.82 ± 2.48	6.10 ± 1.86	−0.571	**0.026**
MFO total muscle (mg/kg muscle/min)	7.23 ± 2.93	7.97 ± 2.76	5.43 ± 2.59	0.935	**<0.001**	6.66 ± 3.18	7.97 ± 2.42	−0.455	0.074
VO_2_max (mL/kg/min)	42.63 ± 10.93	47.12 ± 8.40	31.74 ± 8.47	1.826	**<0.001**	39.10 ± 11.47	47.29 ± 8.25	−0.802	**0.002**
Leptin (pg/mL)	3473.0 ± 3902.9	2100.3 ± 2745.7	6796.6 ± 4340.3	−1.431	**<0.001**	4221.5 ± 4191.3	2484.1 ± 3301.9	0.453	0.075
Log leptin (pg/mL)	3.22 ± 0.59	3.01 ± 0.54	3.71 ± 0.37	−1.408	**<0.001**	3.33 ± 0.59	3.06 ± 0.57	0.476	**0.062**
MedDiet adherence score	6.98 ± 1.68	7.30 ± 1.46	6.21 ± 1.96	0.676	**0.016**	5.78 ± 1.13	8.57 ± 0.63	−2.927	**<0.001**

Values are presented as mean ± standard deviation (SD). NW = normal weight, OW/OB = overweight or obese; MD = mediterranean diet score d = Cohen’s d; BMI = body mass index; MedDiet = Mediterranean diet; MFO = maximal fat oxidation; VO_2_max = maximal oxygen consumption. Statistical significance was set at *p* < 0.05. Bold values indicate statistical significance (*p* < 0.05).

**Table 2:** Association of adherence to the Mediterranean Diet with body mass index, maximal fat oxidation, VO_2_max and leptin levels.

Mediterranean Diet				
	BMI (kg/m^2^)			
	B	β	R^2^	*p*
**Model 0**	−0.864	−0.339	0.115	**0.006**
**Model 1**	−0.871	−0.342	0.116	**0.006**
**Model 2**	−0.473	−0.186	0.374	*0.082*
**Model 3**	−0.483	−0.189	0.376	*0.079*
	Absolute MFO (mg/min)			
	B	β	R^2^	*p*
**Model 0**	0.032	0.338	0.114	**0.006**
**Model 1**	0.030	0.319	0.157	**0.008**
**Model 2**	0.026	0.271	0.163	**0.029**
**Model 3**	0.024	0.255	0.203	**0.037**
	MFO-BM (mg/kg/min)			
	B	β	R^2^	*p*
**Model 0**	0.542	0.395	0.156	**0.001**
**Model 1**	0.537	0.392	0.158	**0.001**
**Model 2**	0.401	0.292	0.273	**0.012**
**Model 3**	0.398	0.290	0.274	**0.014**
	MFO-MM (mg/kg/min)			
	B	β	R^2^	*p*
**Model 0**	0.614	0.352	0.124	**0.004**
**Model 1**	0.629	0.361	0.134	**0.003**
**Model 2**	0.460	0.264	0.209	**0.029**
**Model 3**	0.475	0.273	0.221	**0.025**
	VO_2_max (mL/kg/min)			
	B	β	R^2^	*p*
**Model 0**	3.000	0.462	0.213	**<0.001**
**Model 1**	2.772	0.426	0.365	**<0.001**
**Model 2**	2.151	0.331	0.400	**0.002**
**Model 3**	1.957	0.301	0.541	**0.002**
	Log Leptin (pg/mL)			
	B	β	R^2^	*p*
**Model 0**	−0.100	−0.284	0.081	**0.022**
**Model 1**	−0.081	−0.230	0.447	**0.018**
**Model 2**	−0.079	−0.223	0.077	0.122
**Model 3**	−0.062	−0.176	0.480	*0.074*

Statistically significant results (*p* < 0.05) in the linear regression analyses are highlighted in bold. Statistical trends (*p* = 0.05 ≤ *p* < 0.10) are indicated in italics. The analysis evaluates the association between adherence to the MedDiet and body mass index (BMI), maximal fat oxidation (MFO), MFO per body mass (MFO-BM), MFO per total muscle mass (MFO-MM), VO_2_max, and leptin levels (log-transformed). Abbreviations: *p*, *p*-value; *β*, standardized regression coefficient; *B*, unstandardized regression coefficient; *R^2^*, coefficient of determination; BMI, body mass index (kg/m^2^); MFO, maximal fat oxidation; Leptin, log-transformed leptin concentrations; VO_2_max: maximal oxygen uptake. Models Model 0: unadjusted; Model 1: adjusted for sex; Model 2: adjusted for age; Model 3: adjusted for sex and age.

**Table 3:** Association of leptin levels with body mass index, maximal fat oxidation, and VO_2_max.

Log Leptin				
	BMI (kg/m^2^)			
	B	β	R^2^	*p*
**Model 0**	3.979	0.550	0.302	**<0.001**
**Model 1**	6.563	0.906	0.498	**<0.001**
**Model 2**	3.040	0.420	0.505	**<0.001**
**Model 3**	5.396	0.745	0.648	**<0.001**
	Absolute MFO (mg/min)			
	B	β	R^2^	*p*
**Model 0**	−11.998	−0.650	0.422	**<0.001**
**Model 1**	−11.577	−0.627	0.423	**<0.001**
**Model 2**	−9.963	−0.540	0.569	**<0.001**
**Model 3**	−8.567	−0.464	0.577	**<0.001**
	MFO-BM (mg/kg/min)			
	B	B	R^2^	*p*
**Model 0**	−1.176	−0.302	0.091	**0.014**
**Model 1**	−1.639	−0.421	0.113	**0.008**
**Model 2**	−0.758	−0.195	0.230	*0.098*
**Model 3**	−1.065	−0.273	0.238	*0.075*
	MFO-MM (mg/kg/min)			
	B	β	R^2^	*p*
**Model 0**	−0.589	−0.119	0.014	0.345
**Model 1**	−1.309	−0.264	0.047	0.102
**Model 2**	−0.073	−0.015	0.146	0.905
**Model 3**	−0.610	−0.123	0.162	0.439
	VO_2_max (mL/kg/min)			
	B	β	R^2^	*p*
**Model 0**	−0.063	−0.233	0.054	*0.061*
**Model 1**	−0.037	−0.139	0.068	0.383
**Model 2**	−0.043	−0.160	0.119	0.202
**Model 3**	−0.006	−0.024	0.144	0.883

Statistically significant results (*p* < 0.05) in the linear regression analyses are highlighted in bold. Statistical trends (*p* = 0.05 ≤ *p* <0.10) are indicated in italics. The analysis evaluates the association between leptin levels (log-transformed) and body mass index (BMI), maximal fat oxidation (MFO), MFO per body mass (MFO-BM), MFO per total muscle mass (MFO-MM), and VO_2_max levels. Abbreviations: *p* = *p*-value; β = standardized regression coefficient; B = unstandardized regression coefficient; R^2^ = coefficient of determination; BMI = body mass index (kg/m^2^); MFO = maximal fat oxidation; Leptin = log-transformed leptin concentrations; VO_2_max = maximal oxygen uptake. Model 0 = unadjusted; Model 1 = adjusted for sex; Model 2 = adjusted for age; and Model 3 = adjusted for sex and age.

## Data Availability

The data presented in this study are available on reasonable request from the corresponding author.
